# Regulatory Role of Sex Hormones in Cardiovascular Calcification

**DOI:** 10.3390/ijms22094620

**Published:** 2021-04-28

**Authors:** Holly J. Woodward, Dongxing Zhu, Patrick W. F. Hadoke, Victoria E. MacRae

**Affiliations:** 1 The Roslin Institute & R(D)SVS, University of Edinburgh, Easter Bush, Midlothian EH25 9RG, UK; vicky.macrae@roslin.ed.ac.uk; 2 Guangzhou Institute of Cardiovascular Disease, Guangdong Key Laboratory of Vascular Diseases, State Key Laboratory of Respiratory Disease, the Second Affiliated Hospital, Guangzhou Medical University, Guangzhou 510260, China; 3 University/BHF Centre for Cardiovascular Science, University of Edinburgh, Edinburgh, 47 Little France Crescent, Edinburgh EH16 4TJ, UK; patrick.hadoke@ed.ac.uk

**Keywords:** calcification, testosterone, estrogen, atherosclerosis, aortic valve

## Abstract

Sex differences in cardiovascular disease (CVD), including aortic stenosis, atherosclerosis and cardiovascular calcification, are well documented. High levels of testosterone, the primary male sex hormone, are associated with increased risk of cardiovascular calcification, whilst estrogen, the primary female sex hormone, is considered cardioprotective. Current understanding of sexual dimorphism in cardiovascular calcification is still very limited. This review assesses the evidence that the actions of sex hormones influence the development of cardiovascular calcification. We address the current question of whether sex hormones could play a role in the sexual dimorphism seen in cardiovascular calcification, by discussing potential mechanisms of actions of sex hormones and evidence in pre-clinical research. More advanced investigations and understanding of sex hormones in calcification could provide a better translational outcome for those suffering with cardiovascular calcification.

## 1. Clinical Consequences of Cardiovascular Calcification

Cardiovascular calcification describes the regulated deposition of mineral in blood vessels (vascular calcification) and heart valves (valvular calcification). Calcification is considered a predictor of risk associated with vascular disease [1], with more than 60% of people over 65 years of age displaying calcification in their cardiovascular system [2]. If left untreated, calcification can lead to a number of significant clinical consequences, including coronary insufficiency, aortic stenosis, and, in severe cases, heart failure. Vascular calcification was previously considered the consequence of passive precipitation of calcium and phosphate in the vascular system due to ageing. However, over the past decades, studies have revealed that cardiovascular calcification is indeed an actively regulated process that shares many similarities with physiological bone formation [3]. Despite extensive characterization of cardiovascular calcification in patients, the precise mechanisms that initiate and regulate calcification are still unclear. There is also a distinct sex difference in patients, with males having a tendency to acquire calcification earlier in life, and females developing calcification post-menopause [4]. Although the mechanism(s) behind this sex difference remain(s) to be fully elucidated, current evidence suggests a strong link between the specific actions of individual sex hormones and cardiovascular calcification. In this review, we seek to complement the recent excellent publication by Zhang et al. [5] by discussing the current understanding of the underlying mechanisms through which estrogens and androgens regulate calcification in blood vessels and valves, including a focus on the role for pre-clinical models in this research.

## 2. Types of Cardiovascular Calcification 

**Figure 1:**
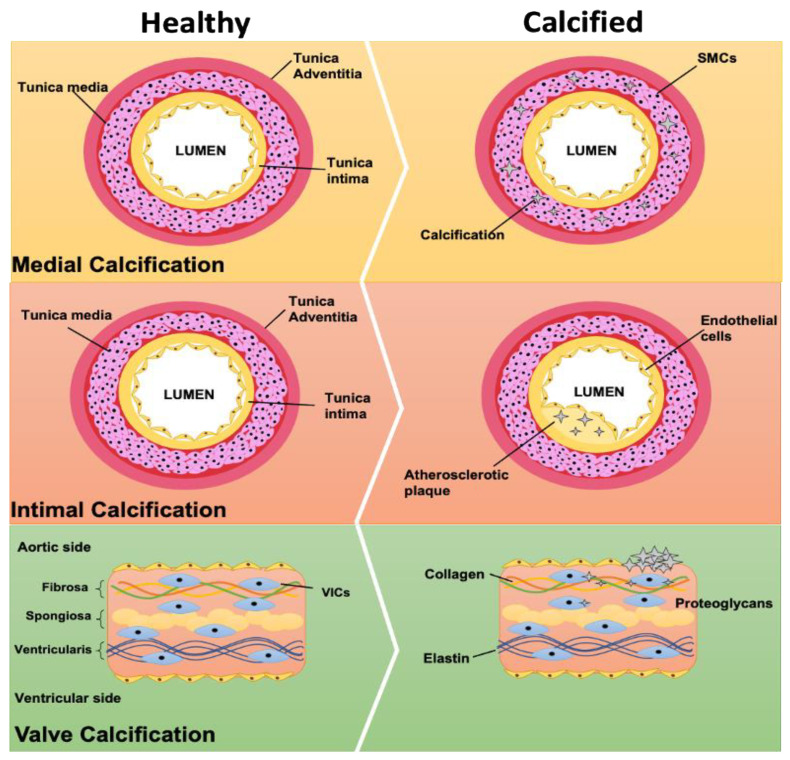
Comparison of types of cardiovascular calcification. The most common types of cardiovascular calcification are medial, intimal and valve. This figure displays both the normal and disease state for each of these diseases. Medial calcification occurs in the tunica media of the aorta, around the smooth muscle cells. Intimal calcification occurs within an atherosclerotic plaque in the intima of the artery. Valve interstitial cells, which could have a role in valve calcification, mostly accumulate in the aortic side of the valve.

According to its location, cardiovascular calcification can be divided into three major types: atherosclerotic intimal vascular calcification, medial vascular calcification, and aortic valve calcification. Within the scientific literature, cardiovascular and vascular calcification terms are frequently used interchangeably, however it is important to recognize that these are separate processes. The following section addresses these different types of calcification in more detail (Figure 1). 

### 2.1. Atherosclerotic Intimal Calcification

There are two types of calcification—intimal or medial—that occur in large arteries. Intimal calcification occurs as a result of atherosclerotic plaque development [6]. During blood vessel calcification, vascular smooth muscle cells (VSMCs) lose their contractile phenotype and become osteogenic, depositing matrix vesicles that enhance calcification [7]. Intimal calcification in atherosclerotic plaques can either stabilise or destabilise the plaque depending on the pattern of deposition and plaque composition. There is evidence that macrocalcification stabilises the fibrous cap covering the atherosclerotic plaque, whereas microcalcification destabilises the plaque, provoking a pro-inflammatory response causing increased susceptibility to plaque rupture [8]. Plaque rupture is considered a major cause of blood vessel stenosis and ischemia in patients, directly leading to thrombus formation and myocardial infarction [9]; although it has been suggested that superficial erosion of plaques may be more significant than plaque rupture as a cause of thrombosis [10]. Within blood vessels, VSMCs in the media, myofibroblasts in the adventitia and pericytes in the micro vessels can also become calcified [11].

### 2.2. Medial Calcification

Medial calcification is linked to altered bone and mineral metabolism and is a clinical predictor of coronary artery disease [2]. Additionally, it is highly prevalent in patients with end stage renal disease (ESRD) and diabetes [12]. Medial calcification occurs in vessels without inflammatory or lipid cell infiltration into the vessel wall and occurs along the elastic fibres of blood vessels causing vessel stiffening and decreased compliance [13]. Medial calcification can also occur in rare diseases such as Mönckeberg’s Arteriosclerosis and Kawasaki disease [14]. The blood vessel stiffening (sclerosis) associated with medial calcification can result in increased blood pressure, local ischaemia and a high risk of vascular mortality [15,16]. Furthermore, medial calcification can lead to heart failure due to loss of aortic elasticity [17]. 

### 2.3. Valvular Calcification 

Calcific aortic valve disease (CAVD) is the most common valve disease in the western world, affects up to 25% of the older population, and currently has no pharmaceutical interventions [18]. The pathogenesis of valve disease is not completely understood and there is a debate as to whether it is a feature of atherosclerosis or is independent of traditional CV risk factors. Endothelial cell disruption and basement membrane damage are evident in early-stage valve disease; one theory proposes that this could be caused by mechanical injury as a result of valve movement [19]. 

Clinically, progression of valve disease is well-characterised. Initial stages involve endothelial cell damage, infiltration of lipids and macrophages, as well as lipid oxidisation [20]. In later stages fibrosis and calcification cause obstruction of the aortic valve opening [20]. Valve interstitial cells (VICs) have been proposed to play a role in valve calcification. Quiescent VICs (qVICs) are the main cell type in a normal valve and function to maintain physiological valve structure and inhibit angiogenesis. If VICs are injured these cells can become activated. These activated VICs (aVICs) have the capacity to adapt to a dynamic environment through their activation and secretion of proteolytic enzymes mediating extracellular matrix remodelling followed by a normalization of phenotype [19]. When VICs are cultured in osteoblastic culture medium, they differentiate to osteoblastic cells (obVICs). Proteins associated with osteogenesis (including osteopontin, bone sialoprotein, alkaline phosphatase and bone morphogenetic protein (BMP)-2 and -4) have been identified by in vitro studies [21]. ObVICs also produce matrix vesicles which deposit calcium in the valves in the form of hydroxyapatite (HA) crystals. Development of macrocalcification and large calcium nodules is extremely damaging in valves. These nodules appear on the aortic side of the valve and restrict valve movement, causing stenosis and aortic regurgitation [22]. 

### 2.4. Pharmaceutical Strategies 

Numerous pharmaceutical interventions against medial and intimal vascular calcification have been interrogated, ranging from statins to phosphate binders, with little therapeutic effect [15]. Vascular calcification is difficult to treat due to calcification having multiple causes, some of which are idiopathic. Furthermore, common diseases such as hypertension, chronic kidney disease, osteoporosis and hyperlipidaemia accelerate the progression of vascular calcification (reviewed in Lu et al. [23]). Therefore, future treatment approaches may need to be personalized, and reflect the other co-morbidities of the patient [24].

There are currently no pharmaceutical interventions for valve calcification. Clinically, severe stenosis can only be treated with either a mechanical or bioprosthetic valve replacement. Without surgery patient prognosis is between 2–3 years [25]. Synthetic valves characteristically have a 20-year lifespan, bringing increased mortality risks for patients requiring subsequent valve replacements [26,27]. Following surgery, patients are typically prescribed anticoagulants and/or immunosuppressants although their effectiveness on clinical outcomes and patient haemodynamic is debated [28]. 

## 3. Current Understanding of Cardiovascular Calcification 

### 3.1. Calcification Is Similar to Physiological Bone Formation 

**Figure 2:**
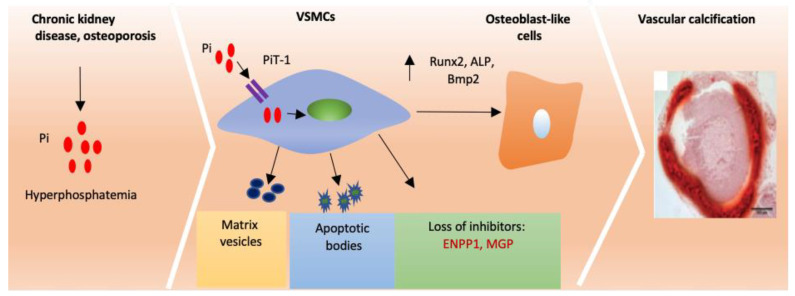
Vascular calcification mechanisms. In individuals with chronic kidney disease (CKD) high serum phosphate induces osteogenic transition and calcification of vascular smooth muscle cells (VSMCs) through the sodium-dependent phosphate cotransporter, Pit-1. In addition, normal blood vessels express a number of calcification inhibitors including ectonucleotide pyrophosphatase/phosphodiesterase 1 (ENPP1) and matrix Gla protein (MGP). Loss of these inhibitors promotes osteogenic transition and calcification of VSMCs. RUNX2, ALP and BMP2 become elevated which promotes osteoblast-like redifferentiation in SMCs. Matrix vesicles and apoptotic bodies also play a role in vascular calcification. Pi-phosphate RUNX2- runt-related transcription factor 2, ALP-alkaline phosphatase Bmp2-bone morphogenic protein 2.

Accumulating evidence shows that cardiovascular calcification is an active, cell-mediated process that shares many similarities with physiological bone formation [29] (Figure 2). Indeed, mature, lamellar bone, with hematopoietic elements and active bone remodelling, has been identified in up to 15% of calcified arteries [16]. Consistent with these findings, bone specific genes (including alkaline phosphatase (*ALP*), osteocalcin (*OCN*), runt-related transcription factor 2 (*RUNX2*), msh homeobox 2 (*MSX2*), and SRY-box transcription factor 9 (*SOX9*)) are significantly induced in calcified human arteries [30]. VSMCs, the predominant cell type in blood vessels, can undergo osteochondrogenic/osteocytic differentiation in the presence of calcifying conditions [31,32]. In vitro studies have demonstrated that high phosphate conditions mimicking the hyperphosphataemia seen in ESRD patients induces VSMC calcification and osteochondrogenic transition [31,33], in which the sodium-dependent phosphate cotransporter PiT1 plays a role. Knockdown of PiT1 expression in VSMCs in vitro significantly reduces phosphate uptake, expression of the osteogenic marker RUNX2, and calcium deposition [33]. 

Bone morphogenic proteins (BMPs) are regulators of physiological bone formation and have also been proposed to regulate vascular calcification. Previous reports, including studies from our laboratory, have shown that serum levels of BMP2 and BMP9 are elevated in patients with ESRD [34,35]. BMP2 enhances high phosphate-induced VSMC calcification in vitro and osteochondrogenic differentiation through up-regulation of RUNX2 [36]. Suppression of phosphate uptake using a sodium-dependent phosphate cotransporter inhibitor, phosphonoformic acid (PFA), attenuates BMP2-induced VSMC calcification in vitro [36]. To note, there is also evidence that PFA can inhibit calcium phosphate deposition independently of phosphate transport, potentially through a physicochemical mechanism [37].

BMP9 induces VSMC osteogenic differentiation and calcification through the orphan activin receptor-like kinase 1 (ALK1)-mediated pSmad1/5/8 signalling pathway [35]. The Wnt/β-catenin pathway is also involved in BMP9-induced VSMC calcification [38]. Consistent with these observations, pharmacological inhibition of BMP signalling reduces vascular calcification in a murine atherosclerotic model [39]. In contrast, BMP7 treatment efficiently reduces arterial calcification in murine models of atherosclerosis and ESRD [40]. 

### 3.2. Loss of Endogenous Inhibitors Induces Vascular Calcification 

VSMCs within normal arteries have been shown to express a number of inhibitors, including matrix Gla protein (MGP) and ectonucleotide pyrophosphatase/phosphodiesterase 1 (ENPP1), which protect against ectopic calcification [19]. MGP is a secreted carboxyglutamic acid modified protein [41]. Mice that lack *Mgp* show arterial calcification [42]. Homozygous loss-of-function of MGP in humans is associated with Keutel’s syndrome, in which patients exhibit excessive calcification of cartilaginous tissues and diffuse arterial calcification [43]. A possible mechanism through which MGP abrogates vascular calcification is by antagonism of BMP signalling. MGP can directly bind to BMP2 and BMP4, thus inhibiting their downstream signalling [44,45]. In accordance with these data, inhibition of BMP signalling using LDN-193189 or ALK3-Fc reduces vascular calcification in *Mgp* null mice [46]. MGP undergoes γ-glutamate carboxylation, in a vitamin-k dependent mechanism, to fully exert its protective effect on arterial calcification [47]. The vitamin K antagonist warfarin rapidly induces arterial calcification through inactivation of MGP in rodents, which can be rescued by a vitamin K-enriched diet [48]. 

ENPP1 is an ecto-enzyme that hydrolyzes ATP to generate pyrophosphate (PPi), which acts as a calcium phosphate crystallization inhibitor when high levels of PPi are produced. ENPP1 is widely expressed in various cells including chondrocytes, osteoblasts and VSMCs [49,50]. Tiptoe-walking (ttw/ttw) mice with a natural occurring nonsense truncation mutation in *Enpp1*, or *Enpp1* null mice, develop extensive peri-articular and arterial calcifications, and progressive ectopic ossification of the spinal ligaments [51,52]. Loss-of-function ENPP1 mutations in humans have been associated with Generalized Arterial Calcification of Infancy (GACI) [53,54]. Functional characterization of these ENPP1 mutations demonstrates a complete loss or partial reduction of its enzymatic activity, thereby reducing the generation of PPi [55]. Beyond the regulation of vascular calcification, ENPP1 also plays a role in the pathogenesis of osteoarthritis, atherosclerosis, insulin resistance and diabetes through mechanisms including the production of high levels of PPi [56,57,58].

### 3.3. Matrix Vesicles and Apoptotic Bodies Promote Cardiovascular Calcification 

Matrix vesicles (MVs) are extracellular membrane-derived microparticles (approximately 100 nm in diameter). MVs have been implicated in bone mineralization, where they serve as the initial nucleation sites for hydroxyapatite formation [59]. Over recent years, the role of MVs in the regulation of cardiovascular calcification has attracted extensive attention. Previous work, including data from our laboratory, has reported the accumulation of MVs in calcified human aortic valves [60], aortic medial tissue, and atherosclerotic plaques [61]. These MVs are released by VSMCs, macrophages and valve interstitial cells, which play a role in initiating vascular calcification [60,62,63]. Proteomic analysis demonstrates that MVs released by cardiovascular cells during calcification show up-regulation of the calcium-binding Annexins but reduced expression of calcification inhibitors, including MGP and fetuin A [60,64]. Of note, vascular cell-secreted MVs contain a large amount of microRNAs, which also play a role in regulating vascular calcification [65]. A number of microRNAs identified in MVs released by vascular cells are involved in osteogenic differentiation. For example, miR-30 identified in MVs regulates the expression of osteogenic genes including *RUNX2* and *SMAD1*, and VSMC calcification [66]. In addition, MVs can be taken up by recipient VSMCs, leading to altered MAPK signalling and calcium metabolism that further drives vascular calcification [67].

Apoptosis, also termed programmed cell death, is a tightly regulated, energy dependent process which is mainly regulated by caspases [68]. Apoptotic cells exhibit blebbing, cell shrinkage, and nuclear and DNA fragmentation. Previous studies have shown apoptosis to increase during VSMC [69] and VIC calcification [70] in vitro. Induction of apoptosis using anti-Fas IgM and cycloheximide enhances VSMC calcification, while inhibition of apoptosis using the caspase inhibitor z-VAD-FMK attenuates VSMC calcification in vitro [69]. Furthermore, activation of the Gas6/Axl/Akt survival pathway reduces VSMC calcification [71]. PiT1 is required for high phosphate-induced apoptosis in both VICs [72] and VSMCs [73]. Mechanistically, apoptotic bodies may expose phosphatidylserine on the outer membranes and generate a potential calcium-binding site suitable for HA deposition [69]. In this review we have focused on activation of calcification but it is important to note that calcification is a balance of both continuous activation and lack of resolution of calcification (reviewed in Carracedo et al. [74]).

## 4. The Role of Sex and Sex Hormones in Cardiovascular Calcification

### 4.1. Sex difference Exists in Cardiovascular Calcification

Sex differences and effects of sex hormones have been associated with cardiovascular calcification. Men have a 2-fold increased risk, compared with women, of developing CAVD [75]. Clinical studies of CAVD have demonstrated that for the same degree of stenosis, male patients have more calcification than females, whereas female patients have more fibrosis [76,77]. Furthermore, studies have shown that valve disease does not develop until after menopause in women [4].

Males also develop vascular disease, including atherosclerosis, earlier than females [78]. Circulating levels of testosterone, the predominant sex hormone in men, are positively associated with vascular calcification [79]. In elderly male patients with stable coronary artery disease, serum testosterone levels are inversely associated with vascular calcification [80]. Additionally, females with polycystic ovary disease (PCD) develop elevated levels of testosterone and have an increased risk of cardiovascular disease [81]. Over the past decades, it has become accepted that women are protected against cardiovascular disease; however, these protective effects are lost post-menopause. The most established hormonal change in postmenopausal women is the decline of estrogen levels [82], which is considered a causal factor for the increased incidence of vascular disease [83]. Serum levels of estrogen are negatively associated with vascular calcification [84]. Whilst established dogma highlights testosterone as a risk factor for calcification and estrogen as cardioprotective, the specific pathways underpinning how these sex hormones induce calcification have yet to be fully elucidated. Our current knowledge on the mechanisms through which sex hormones and their receptors interact with calcification, is summarised below.

### 4.2. Estrogen and Activation of the Estrogen Receptor Prevents Calcification

Clinical studies have demonstrated that estrogen replacement therapy (ERT) reduces cardiovascular disease in postmenopausal women [85] and that the effectiveness of ERT may be dependent on the “timing” of estrogen delivery relative to the age of menopause onset (within 6 years of menopause onset) [86]. Recently, the controlled Women’s Health Initiative Study reported that long-term estrogen therapy reduced vascular calcification in postmenopausal women aged 50 to 59 years [87], consistent with a protective role. Studies employing estrogen replacement in ovariectomy-induced vascular calcification and osteoporosis in *Apoe*^−/−^ mice further corroborate these data [88].

**Figure 3:**
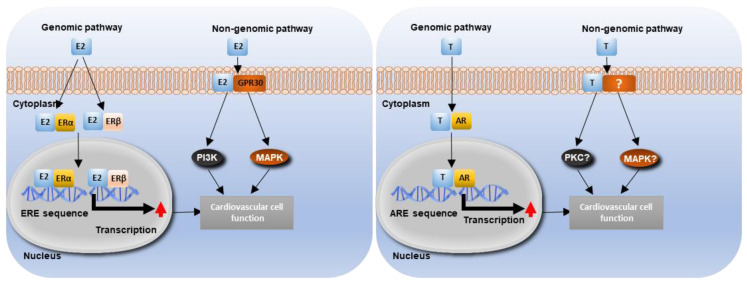
Estrogen binds to estrogen receptors (ERs), which act as transcription factors to regulate the expression of genes containing estrogen response elements (ERE). In addition, estrogen also rapidly induces phosphoinositide 3-kinase (PI3K) and mitogen-activated protein kinase (MAPK) signaling through the orphan G-protein coupled receptor (GPR30). Through genomic and non-genomic pathways, estrogen regulates a wide range of vascular cell functions, including proliferation, migration and senescence. E2- Estradiol. Testosterone acts in a similar mechanism, binding to the androgen receptor (AR), which act as transcription factors to regulate the expression of genes containing the androgen response elements (ARE). Testosterone is also believed to have non-genomic effects, although these pathways are less clear than estrogens.

In VSMCs, the expression of estrogen receptor α (ERα) is greater than that of estrogen receptor β (ERβ). Estrogen mainly acts through ERα (Figure 3) to inhibit RANKL signalling, thereby reducing the osteogenic differentiation and calcification of VSMCs by upregulating BMP and downregulating MGP [88]. In addition, estrogen can inhibit vascular calcification through regulation of a range of molecular and cellular events, including hypoxia-induced factor-1α signalling [89], autophagy [90] and estrogen receptor α-dependent growth arrest-specific gene 6 transactivation [91]. Conversely, exogenous estrogen application has been reported to enhance vascular calcification in bovine aortic medial cells (in vitro) [92] and in aged male and female *ApoE^−/−^* mice (in vivo) [93]. These studies suggest a complex role of estrogen in vascular calcification. Estrogen can be generated in blood vessels by aromatase-mediated conversion of testosterone [94] and therefore, the activity of aromatase in blood vessels may also have a role in mediating the effects of estrogen on vascular calcification and warrants future investigation.

### 4.3. Testosterone Is a Risk Factor for Cardiovascular Calcification

Low levels of circulating testosterone have been associated with increased coronary artery calcification in non-obese Korean men [95]. However, the recent Framingham Heart Study reports no significant association between testosterone and vascular calcification after model adjustment for other vascular risk factors in community-dwelling men [96]. In post-menopausal women, higher free serum testosterone is associated with coronary artery calcium progression [97]. Experimental studies show conflicting results on the effect of testosterone in calcification. In both male and female *Apoe*^−/−^ mice, testosterone administration increases atherosclerotic calcification, which involves both androgen receptor- and estrogen receptor-mediated pathways [98]. Consistent with these results, our laboratory has previously shown that deletion of androgen receptor in VSMCs prevents testosterone-induced VSMC calcification in vitro [99]. Contrasting studies report an inhibitory effect of testosterone on VSMC calcification, which is mediated through androgen receptor-dependent transactivation of growth arrest-specific gene 6 signalling [100]. This discrepancy may reflect the complex actions of testosterone within different in vitro models. Clinical studies have shown that, in men, low endogenous testosterone levels are associated with cardiovascular disease [101] but also that high testosterone (for example with anabolic steroid use) has adverse cardiovascular effects [102]. This type of U-shaped relationship has been recognized with other hormones: for example, with corticosteroids where insufficiency causes Addison’s disease but excess causes Cushing’s syndrome [103].

## 5. Sex Hormones Mediate Cellular Signalling Pathways in the Cardiovascular System

### 5.1. Estrogen Signalling and Cardiovascular Function

Estrogen regulates cardiovascular function through several ERs, including ERα, ERβ and the orphan G-protein-coupled receptor (GPR30) (Figure 3) [104,105]. Upon binding to estrogen, ERs undergo conformational change, leading to ER dimerization and binding to consensus (5′GGTCAnnnTGACC 3′) estrogen response element (ERE) sites on nuclear DNA. Co-regulators are then recruited to the estrogen-ER complex to activate or inhibit gene expression [106]. Estrogen also has the capacity to regulate gene expression indirectly by altering the activity of other transcription factors (such as AP1 and Sp1) [107,108]. In the absence of estrogen, ER at specific serine sites can be phosphorylated by Epidermal Growth Factor (EGF), thereby mediating the stimulatory effects of EGF [109]. In addition to its genomic action, estrogen also induces acute non-genomic signalling pathways. Estrogen can bind to cell membranes to rapidly induce PI3K signalling. Furthermore, estrogen activates PI3K and MAPK through binding to GPR30 [110]. Functional classical ERs and non-classical GPR30 are widely expressed by vascular cells, including endothelial cells, VSMCs and cardiomyocytes [111]. Through estrogen’s genomic and non-genomic mechanisms, regulation of a wide range of cardiovascular processes has been reported, such as: cardiac hypertrophy and failure, ischemic heart diseases, vascular injury and atherosclerosis [112,113].

### 5.2. Testosterone Signalling and Cardiovascular Function

Testosterone can regulate a range of cardiovascular functions and phenotypes, including vasodilation and vasoconstriction and intima-media thickness [114]. Similar to estrogen, testosterone induces both genomic and non-genomic actions. Testosterone binds to the androgen receptor (AR) to regulate gene expression through binding with the androgen response element (ARE) [115]. It also induces rapid non-genomic effects through binding to membrane androgen receptors or sensors, leading to the activation of a range of intracellular signalling molecules including calcium (Ca^2+^), nitric oxide (NO), PKA, PKC, and MAPK [116]. The AR is expressed in several vascular cell types, including VSMCs and endothelial cells [117], and has been shown to be by expressed within the aortic valve (although the identity of valve cells expressing AR remains unclear). Testosterone can also be converted to 5α-dihydrotestosterone (DHT; which has a higher binding activity to the AR) by the cytochrome P-450 enzyme, 5α-reductase [118]. As testosterone can be converted into estrogen through aromatase, AR activation is also regulated by the cell-specific profile of metabolic products of testosterone [94]. Aromatase has been observed in vascular endothelial cells and 5α-reductase has been shown to be expressed in VSMCs [119,120]. Aromatase expression has been observed in the aortic sinus of mice, but the cell-type expressing the enzyme is unknown [98].

## 6. Animal Models Offer Insights into Sex Differences in Cardiovascular Calcification

Females are typically under-represented in pre-clinical studies. Indeed, there is concerning evidence that within biological and medical research, 80% of studies that specified sex used only male subjects [121]. This lack of inclusion of female models likely contributes to the poorer treatment outcomes for women and a reduced understanding of sex differences in the calcification process [122]. In this section we discuss the use of both male and female in vivo models in calcification research.

### 6.1. Rodent Models

The human cardiovascular system comprises a complex arrangement of specialized structures with distinct functions. The current use of small rodents as the main model of human diseases is widespread. They are relatively cost effective and easy to maintain. Furthermore, rodent models offer opportunities for genetic manipulation and pre-clinical imaging techniques, making them indispensable for elucidating the mechanisms underpinning cardiovascular disease [123]. Nonetheless, limitations do exist, and significant vascular differences are apparent between humans and rodents.

Rodent models are highly resistant to vascular calcification. In order to investigate cardiovascular calcification in rodents, the pathological process has to be artificially induced, typically through diet, genetic manipulation or mechanical injury [124]. The most commonly used model of vascular and valvular calcification is the *Apoe*^−/−^ mouse model fed a “western” diet [125]. However, calcification does not present uniformly across all animals within a cohort. Additional genetic models of calcification include mice lacking low density lipoprotein receptor (*Ldlr*) (intimal calcification) [126], *Enpp1*(medial) [127], *Mgp* (medial) [42] and ATP binding cassette subfamily C Member 6 (*Abcc6*) (medial) [128,129]. These models primarily display aortic calcification but valvular calcification has also been observed in the *Ldlr*^−/−^ and *Apoe*^−/−^ mice [130]. Vascular calcification is a clinical consequence of ESRD, therefore it is reassuring that many models of this condition (including dietary addition of adenine, vitamin D administration and surgical induction of kidney insufficiency through 5/6 nephrectomy) have also been developed to interrogate the process of vascular calcification [131].

Exposure of rats to warfarin also produces calcification in the aorta and aortic valves [132]. However, inconsistent levels of calcification are typically observed in these rodent models, highlighting the requirement for more refined options [133].

A number of approaches can be employed to investigate the sex hormone effect on cardiovascular calcification in vivo. Sex hormone receptors can be ablated; the *Ar*^−/−^ mouse has been utilized to investigate the effect of testosterone in vascular calcification [99]. Furthermore, studies involving mice lacking ERα and ERβ, have elucidated that these receptors are necessary for estrogen-mediated inhibition of the vascular injury response [134]. A physiological approach to investigating the effects of sex hormone involves the surgical removal (ovariectomy or castration) of sexual organs. Indeed, ovariectomised mice have been used to investigate bone calcification and show impaired bone formation [135].

Interestingly, sex differences in the atherosclerotic phenotype observed in *Apoe*^−/−^ mice have been well-defined, whereby females tend to have larger plaques and increased calcification in those plaques [136]. Conversely, in human patients, females typically have smaller plaques and less calcification within the plaques [137]. Whilst investigations are frequently limited by the focus on a single sex, those rodent studies that have employed males and females to interrogate cardiovascular calcification pathways (Table 1), have produced divergent findings; highlighting the constraints of these animal models, and the importance of investigating both sexes in pre-clinical studies.

### 6.2. Large Animal Models

An inherent resistance to the development of cardiovascular calcification offers a crucial limitation to the employment of small animal models to investigate pathways underpinning this pathological process [146,147]. Subsequently novel therapeutic interventions for vascular or valvular calcification require pre-clinical testing in both small and large animal models to assess their suitability for clinical application.

Rabbits are a frequently employed, and highly appropriate, animal model of valvular calcification, due to the tri-layered composition of their valve leaflets (similar composition to humans) [148]. Methods of inducing calcification in rabbits include diet (e.g., vitamin D and long-term cholesterol treatment), genetic manipulation (e.g., LDLR mutated Watanabe rabbits), and surgical intervention (e.g., aortic balloon injury) [149,150,151,152]. However, studies have typically focused on male rabbits, with an extremely limited number of calcification studies including female animals. A recent study assessing both sexes in a matrix metallopeptidase 9 (MMP-9) overexpression model of atherosclerosis, revealed that both male and female rabbits developed calcified lesions in the aortic arch, with qualitative assessment suggesting greater calcification in males [153].

Porcine and ovine models are commonly used in investigations of aortic stenosis, with the majority of these studies examining synthetic or bioprosthetic valve replacements. An advantage of these larger animal models is the relative size of the vascular structures compared to humans, which permits the implementation of surgical procedures routinely employed in the clinic. Indeed, porcine valves have been an established option for human valve replacement for over 20 years [154]. Porcine models are highly appropriate due to the closely comparable valve anatomy, haemodynamic profiles and lipid composition to human patients [155]. They also show age-dependent insulin resistance [155] and are susceptible to the development of calcified plaques through exposure to a high fat diet [156]. The fibrosa layer in porcine aortic valves has similar features to the human fibrosa, including regions rich in collagen, elastin and proteoglycans [157]. As in humans, porcine aortic valves are more vulnerable to calcification on the aortic side, with molecular investigations confirming that valve endothelial cells (VECs) derived from the aortic side express osteogenic regulators including BMP4 [158]. Indeed, porcine VICs have been used to elucidate genetic sexual dimorphism, with recent microarray analysis of bovine cells highlighting the over-representation of cell pathways including cell death, proliferation, cell-to-cell signalling and movement in male-derived VICs compared to female-derived VICs [159,160]. Future aortic calcification studies are, however, required to confirm these findings in vivo. Due to their size and husbandry needs, when compared to smaller models, employing large animals will involve higher costs. Despite this, their importance in the field of human diseases is evident and will undoubtedly illuminate new biological pathways and mechanisms to facilitate the refinement of therapeutic strategies against cardiovascular calcification.

## 7. Future Perspectives

Despite testosterone being an established risk factor for cardiovascular calcification (and many other vascular diseases) its clinical impact is unclear and its mechanism of action in cardiovascular calcification remains to be fully understood. Whilst females are believed to be ‘cardio-protected’, pre-menopausal patients are severely under-represented in pre-clinical studies, which may contribute to our lack of understanding of the mechanisms underpinning the cardioprotective role of estrogens. Whether these sex hormones directly contribute to the increased vascular calcification observed in postmenopausal women remains to be investigated. Furthermore, elucidating these mechanisms is hampered not only by the limitations of pre-clinical models, but also by the severe under-representation of the female sex in pre-clinical research. Further research is essential to bridge the knowledge gap between the cellular mechanisms of calcification and the clinical sex risk factors, in order to ensure equitable treatment approaches for patients of both sexes.

## Figures and Tables

**Table 1:** Sex differences in rodent cardiovascular calcification studies. This table summarises key findings of cardiovascular calcification studies that have investigated both sexes, including the model and sex difference. In mice, increased calcification has typically been reported in females compared to males whereas in rats lower calcification has been observed in females. ApoE—Apolipoprotein E, DHT—Dihydrotestosterone, ↑—increased.

Model	Method of Calcification Induction	Type of Calcification	Sex Differences in Calcification	Treatment	Ref
**Mouse**					
ApoE^−/−^	Aged to 36 weeks	Vascular and valvular	↑ calcification in females (aortic sinus)	17β-estradiol	[93]
ApoE^−/−^	Crossed with *Itga8^−/−^*	Vascular	↑ calcification in females		[93]
*Klotho^−/−^*		Vascular	None	MicroRNA-145 and microRNA-378a	[138]
*Klotho^−/−^*		Vascular	None	None	[139]
ApoE^−/−^		Vascular, aortic sinus	↑ calcification in females	Testosterone and DHT	[98]
ApoE^−/−^	18 months	Vascular	More vascular calcification in males		[140]
ApoE^−/−^	Uraemia	Vascular	↑ calcification in females		[141]
C57BL/6J		Vascular	None		[141]
ApoA-II		Vascular	None		[141]
ApoE^−/−^		Vascular	↑ calcification in females		[141]
ApoE^−/−^	Hyperlipidaemic diet	Vascular	↑ calcification in females (medial arteries)		[142]
**Rat**					
Fisher	1α-Hydroxyvitamin D3	Vascular	↑ calcification in males		[143]
Wistar		Vascular	↑ calcification in males		[144]
Lewis Polycystic Kidney		Vascular	None	Perindopril	[145]
